# Application of symbolic play test in identification of autism spectrum disorder without global developmental delay and developmental language disorder

**DOI:** 10.1186/s12888-023-04647-6

**Published:** 2023-03-06

**Authors:** Xuening Chang, Lingli Sun, Ruizhen Li

**Affiliations:** Department of Child Health Care, Wuhan Children’s Hospital (Wuhan Maternal and Child Healthcare Hospital), Tongji Medical College, Huazhong University of Science & Technology, Wuhan, 430016 China

**Keywords:** Autism spectrum disorders, Symbolic play test, Developmental language disorder

## Abstract

**Background:**

Children with autism spectrum disorders (ASD) usually experience difficulty regarding symbolic play. However, studies on whether symbolic play test (SPT) can differentiate between ASD and other developmental disorders are inconsistent, and evaluating the application value of the SPT in the identification of ASD without global developmental delay (GDD) and developmental language disorder (DLD) is necessary.

**Methods:**

A total of 200 children were selected as the research participants. There were 100 cases of ASD without GDD and 100 cases of DLD. All children were tested by SPT and Children Neuropsychological and Behavioral Scale-Revision (CNBS-R2016). Binomial logistic regression was used for multivariate analysis. The receiver operating characteristic (ROC) curve was used to evaluate the value of SPT in identifying ASD without GDD and DLD.

**Results:**

SPT equivalent age was lower than chronological age in the two groups, the difference between the ASD without GDD group was greater than that in the DLD group, and the proportion of SPT equivalent age retardation was higher than that in the DLD group; the differences were statistically significant. Logistic regression analysis showed that there was a difference in SPT equivalent age between DLD and ASD without GDD. When the cut-off value of the SPT was 8.5, the largest area under the ROC curve was 0.723, and the sensitivity and specificity for the diagnosis of ASD without GDD were 0.720 and 0.620 respectively.

**Conclusions:**

Symbolic play ability in ASD children is worse than that of DLD children at comparable development levels. SPT may be helpful to distinguish ASD without GDD from children with DLD.

## Background

Autism spectrum disorder (ASD) in the DSM is classified as a neurodevelopmental disorder characterized as social communication disorders, narrow interest, or repeated stereotyped behaviors [1]. The incidence of ASD has risen sharply in recent years. The prevalence of ASD has reached 1/44, according to the data of the US Centers for Disease Control and Prevention in 2021 [2]. The male-female ratio was 4.3:1 [3].

Symbolic play arises between 18 and 24 months of life [4]. Symbolic play test (SPT) mainly observes children’s ability to express their self-experience and imagination with the simplest symbols [5]. Some studies indicate that social tendencies, shared attention, and lack of symbolic play are the core deficits of ASD [6]. Children with ASD usually experience difficulty with symbolic play [7].

Research shows the existence of a remarkable relationship between child play and the development of language [8, 9] in children with both typical and atypical development. The delay in the development of language and receptive language predicts the difficulties of children with ASD in pretend play [10].

Language disorders are the most obvious features of children with ASD and also are the direct reason for their hospital visits [11]. Developmental language disorder (DLD) refers to when a child’s language skills are substantially and quantitatively lower than their peers while excluding ASD, global developmental delay (GDD), etc. [12]. ASD usually has some comorbidities, such as GDD [13]. GDD is defined as a delay in two or more developmental domains of gross/fine motor, speech/language, cognition, social/personal, and activities of daily living, affecting children under the age of 5 years [14, 15]. ASD without GDD and DLD are often confused with each other in the early stage of the disease and are difficult to identify [16]. In China, most studies of autism have only focused on children with autism who have intellectual disability (ID) [17]. This is despite the fact that 75% of individuals living with autism spectrum disorder have no intellectual disability [18]. Children with GDD often meet diagnostic criteria for ID and probably represent the same population [19].

Studies have shown that early screening for ASD is far more cost-effective than comprehensive diagnostic assessment without screening [20]. At present, the commonly used screening tools in clinical are the Modified Checklist for Autism in Toddlers (M-CHAT) and the Autism Behavioral Scale (ABC), but both of them are subjectively influenced by their parents, and the screening efficacy is poor [21, 22].

SPT is considered an early indicator for the diagnosis of autism [23] and its assessment [24]. Some studies [25, 26] found that children with ASD present less symbolic play than children with other neurodevelopmental disorders. In contrast, some studies [24, 27] did not find differences between the groups of children with ASD and other neurodevelopmental disorders.

The primary objective of the current study was to provide scientific evidence for the early detection of ASD by evaluating the application value of the SPT in children with ASD without GDD.

## Methods

### Participants

Two hundred children aged 1.5–4.5 years old who were diagnosed with ASD without GDD and DLD in the Child Health Department of Wuhan Children’s Hospital from August 2021 to December 2021 participated in this study. 100 children with ASD without GDD were enrolled, including 79 boys and 21 girls. Their mean chronological age is 28.45 ± 5.34 months. 100 children were diagnosed with DLD, including 87 boys and 13 girls with a mean chronological age of 27.33 ± 5.22 months. There were no statistical differences in the gender distribution (*χ*^*2*^ = 2.268, *P* = 0.132) and chronological age (*t* = 1.498, *P* = 0.136) in these two groups of children. All the clinicians participating in the study were uniformly trained, and all scale assessments were completed by the same professional. The examiner was blind to the participants’ group when completing the assessment. This study was approved by the medical ethical committee of Wuhan Children’s Hospital, Tongji Medical College, Huazhong University of Science and Technology (NO.2021R181). Informed and signed consent was obtained from all parent(s)/caregiver(s) of the participating children. All methods in the present study were performed following the relevant guidelines and regulations of the Declaration of Helsinki.

### Procedure

The enrollment criteria for children with ASD without GDD: ①met the diagnostic criteria of the Diagnostic and Statistical Manual of Mental Disorders, 5th edition (DSM-5) [28], and were diagnosed by 2 deputy chief physicians and above specialists. The Autism Diagnostic Observation Schedule, Second Edition (ADOS-2) and Children Neuropsychological and Behavioral Scale-Revision (CNBS-R2016) were conducted by one professional reviewer; ②no more than one functional domain with a Developmental Quotient (DQ) < 70 in the evaluation results of the CNBS-R2016 [29]. Exclusion criteria: ①Fragile X Syndrome, tuberous sclerosis, cerebral paralysis, epilepsy, schizophrenia, mood disorders, hearing impairment;②abnormal karyotype, appearance deformities, Brain MRI and EEG abnormalities, and other neurological or somatic diseases. The enrollment standard for children with DLD: ①met the diagnostic criteria for DSM-V; ②DQ of language ≤85, DQ of gross motor, fine motor, adaptability, and social behavior ≥70 [30]. ASD, developmental delay, genetic differences, vision, hearing abnormalities, central nervous system disorders, and second language development disorders were excluded [31].

### Instruments included in the study

#### Symbolic play test (SPT)

Four sets are used to assess children’s spontaneous play behaviors in structured situations for a total of 10–15 min of play (3 min per set) with the professional investigator. The investigator cannot use verbal or other communication types to explain the play but can remind children to draw attention to a neglected toy. Four sets of miniature objects are presented in a specific order in different situations, enabling the investigator to observe if the child relates to them appropriately. Objects are purposefully chosen and can lend themselves to a variety of interrelationships. The scoring system is based on the number of meaningful responses and connections the child is able to make.

The scale was made up of 24 items. Set 1 evaluates the following items: Discriminate handing of doll; relates spoon to cup or saucer; feeds, combs, or brushes doll; feeds, combs or brushes the other person; places the cup on the saucer. Set 2 evaluates the following items: Discriminate handing of doll; relates doll to bed; relates blanket or pillow to doll; puts doll to bed; uses pillow correctly. Set 3 evaluates the following items: Relates knife or fork to plate; relates fork, knife, or plate to table; relates tablecloth to other objects; plates doll on the chair; relates fork, knife, or plate to doll; relates chair to table; relates doll to table; places tablecloth on the table. Set 4 evaluates the following items: Moves tractor or trailer along; relates logs to the tractor, trailer or man; places man in the tractor or trailer; places man in driver’s seat; lines up the tractor and trailer; attaches tractor or trailer.

Completion of each item counts as 1 point, otherwise, counts as 0 points, and the final score is the sum of 24 items. Both raw score and age equivalences were provided by the scale. No corresponding age in months was found when SPT raw score is less than 5 or greater than 23. The test has good reliability (Cronbach α = 0.90) and construct-related validity (*r* = 0.77) [5].

### Children neuropsychological and behavioral scale-revision (CNBS-R2016)

CNBS-R2016 evaluates the neurodevelopmental levels and autism symptoms of children. The development quotients (DQs) of the six domains of CNBS-R2016 include gross motor, fine motor, adaptive behavior, language, personal-social, and autism warning behavior. The results are expressed by DQ. DQ greater than 80 is interpreted as the absence of neuropsychological and/or behavioral difficulties. DQ between 70 and 79 indicates a borderline deficiency, and DQ less than 70 indicates a developmental delay. The CNBS-R2016 and the Griffiths Mental Development Scales for China showed good consistency in the developmental assessment of children with ASD [29].

### Autism diagnostic observation schedule, second edition (ADOS-2)

The Autism Diagnostic Observation Schedule 2nd Edition [32] is a standardized semi-structured interview recommended for the assessment of ASD, generally lasting from 30 to 60 min. It includes a range of questions and activities designed to evoke behaviors and cognitions associated with ASD. These visible behaviors and discussions are then scored from 0 to 3 for “autism severity”. Under the original algorithm, 11 items from the larger scoring matrix are then summed to create an ADOS score, where 7 is the cut-off for being designated as “on the autism spectrum,” and 10 is the cut-off for being designated as “autistic.” The algorithm has two subscales: social affect and restrictive and repetitive behaviors, and total scores of 8 or more indicate possible ASD.

### Statistical methods

Continuous variables were described as mean ± standard deviation (SD) and tested with a rank-sum test; categorical variables were described as the proportion (%) and tested with a chi-square test. Finally, multiple logistic regression was used to assess the association between SPT and two groups, while adjusting for confounders. SPT was the dependent variable; disease categories were the primary independent variable, and chronological age, sex, gross motor function, fine motor function, adaptive behavior function, language function, and personal-social function were confounding variables. The odds ratios (*OR*) and 95% confidence intervals (*CI*) were estimated from the multiple logistic regression models. The receiver operating characteristic (ROC) curve was used to analyze the cut-off value of SPT in screening for ASD without GDD. All *P* values were two-tailed with a significant level of 0.05. Statistical analyses were carried out using SAS version 9.4 (SAS Inc., Cary, NC).

## Results

### Comparison of chronological age and SPT equivalent age in the two groups

SPT equivalent age lagged behind chronological age in the two groups, and the differences were statistically significant. The difference between chronological age and SPT equivalent age in ASD without GDD group was greater than that in the DLD group, and the differences were statistically significant (*P* < 0.05) (Table 1).

**Table 1:** Comparison of chronological age and SPT equivalent age in ASD without GDD group and DLD group

project	18–24 months		*P*	ES	25–31 months		*P*	ES	≥32 months		*P*	ES
	N	^−^x ± s			N	^−^x ± s			N	^−^x ± s		
ASD without GDD group												
Chronological age	32	22.27 ± 2.01	< 0.001	3.553	40	28.75 ± 1.70	< 0.001	4.431	28	35.09 ± 2.14	< 0.001	3.961
SPT equivalent age	16	14.50 ± 2.35			34	16.56 ± 3.50			26	19.52 ± 5.13		
DLD group												
Chronological age	31	22.11 ± 1.92	< 0.001	1.517	43	26.87 ± 1.48	< 0.001	1.525	26	34.32 ± 3.82	< 0.001	2.002
SPT equivalent age	27	17.39 ± 3.96			41	20.78 ± 5.45			24	23.49 ± 6.63		

*ASD* Autism spectrum disorders, *GDD* Global developmental delay, *SPT* Symbolic play test, *DLD* Developmental language disorder, *ES* Effect size

### Comparison of symbolic abilities and developmental levels in the two groups

In the 18–24 months group, the proportion of SPT equivalent age seriously lagging (SPT equivalent age is less than 12 months) in the ASD without GDD group was higher than that in the DLD group. The adaptive behavior function, language function, and personal-social function of ASD without the GDD group lagged behind that of the DLD group, and the differences were statistically significant (*P* < 0.05) (Table 2).

**Table 2:** Comparison of symbolic abilities and development levels in ASD without GDD group and DLD group in children aged 18–24 months

project	18–24 months				*χ*^*2*^/t	*P*	ES
	ASD without GDD group		DLD group				
	(*N* = 32)		(*N* = 31)				
SPT equivalent age							
< 12 months	16	80.00%	4	20.00%	10.001	0.002	0.398
≥12 months	16	37.21%	27	62.79%			
Gross motor function	100.09 ± 7.62		98.97 ± 5.68		0.664	0.509	0.167
Fine motor function	89.06 ± 13.40		92.55 ± 9.65		−1.182	0.242	0.299
Adaptive behavior function	86.73 ± 12.45		100.65 ± 11.15		−4.667	< 0.001	1.178
Language function	58.67 ± 11.89		67.48 ± 9.57		−3.236	0.002	0.816
Personal-social function	69.14 ± 7.09		73.48 ± 5.11		−2.782	0.007	0.702

*ASD* Autism spectrum disorders, *GDD* Global developmental delay, *SPT* symbolic play test, *DLD* Developmental language disorder, *ES* Effect size

There are statistically significant differences in the Language function and Personal-social function variables in the 25–31 months group (Table 3). Statistically significant differences are observed in the Fine motor function variable in the ≥32 months group (Table 4). In both the 25–31 months and ≥ 32 months groups, the Adaptive behavior function of ASD without GDD group lagged behind that of the DLD group, and the differences were statistically significant (Tables 3 and 4).

**Table 3:** Comparison of symbolic play ability and development level in ASD without GDD group and DLD group in children aged 25–31 months

project	25–31 months				*χ*^*2*^/t	*P*	ES
	ASD without GDD group		DLD group				
	(*N* = 40)		(*N* = 43)				
SPT equivalent age							
< 12 months	6	75.00%	2	25.00%	1.499	0.221	0.134
≥12 months	34	45.33%	41	54.67%			
Gross motor function	93.95 ± 7.57		94.05 ± 7.27		−0.059	0.953	0.013
Fine motor function	84.30 ± 8.63		89.91 ± 15.87		−1.979	0.051	0.439
Adaptive behavior function	82.08 ± 8.87		94.67 ± 9.34		−6.290	< 0.001	1.382
Language function	61.19 ± 13.65		66.77 ± 9.80		−2.150	0.035	0.470
Personal-social function	69.54 ± 7.50		74.05 ± 5.69		−3.099	0.003	0.678

*ASD* Autism spectrum disorders, *GDD* Global developmental delay, *SPT* symbolic play test, *DLD* Developmental language disorder, *ES* Effect size

**Table 4:** Comparison of symbolic play ability and development level in ASD without GDD group and DLD group in children aged ≥32 months

project	≥32 months				*χ*^*2*^/t	*P*	ES
	ASD without GDD group		DLD group				
	(*N* = 28)		(*N* = 26)				
SPT equivalent age							
< 12 months	2	66.67%	1	33.33%	0.000	1.000	0.000
≥12 months	26	50.98%	25	49.02%			
Gross motor function	92.46 ± 7.92		93.50 ± 6.27		−0.530	0.598	0.146
Fine motor function	78.89 ± 6.36		85.38 ± 9.65		−2.940	0.005	0.794
Adaptive behavior function	79.21 ± 7.32		87.38 ± 9.48		−3.560	0.001	0.965
Language function	64.89 ± 10.96		61.65 ± 8.63		1.201	0.235	0.328
Personal-social function	72.21 ± 6.53		73.12 ± 6.26		−0.517	0.607	0.142

*ASD* Autism spectrum disorders, *GDD* Global developmental delay, *SPT* symbolic play test, *DLD* Developmental language disorder, *ES* Effect size

### The effect of SPT on the differentiation of ASD without GDD and DLD

Logistic regression analysis was performed with disease categories as dependent variables and chronological age, sex, gross motor function, fine motor function, adaptive behavior function, language function, personal-social function, and SPT equivalent age as independent variables. The results showed that there was a difference in SPT equivalent age between DLD and ASD without GDD (*OR* = 1.16, 95%*CI* = 1.06 ~ 1.27) (Table 5).

**Table 5:** Binomial logistic regression analysis of influencing factors on the differentiation of ASD without GDD and DLD

Factor	Estimate of parameter	Standardization of parameter estimates	Chi-square	*P* value	*OR* (95% *CI*)
Sex	0.148	0.564	0.069	0.793	1.16 (0.38–3.50)
Chronological age	−0.209	0.104	4.060	0.044	0.81 (0.66–0.99)
Gross motor function	−0.166	0.104	2.540	0.111	0.85 (0.69–1.04)
Fine motor function	−0.043	0.067	0.406	0.524	0.96 (0.84–1.09)
Adaptive behavior Function	0.301	0.088	11.534	0.001	1.35 (1.14–1.61)
Language function	0.048	0.071	0.468	0.494	1.05 (0.91–1.21)
Personal-social function	−0.036	0.088	0.167	0.682	0.96 (0.81–1.15)
SPT equivalent age	0.150	0.045	11.332	0.001	1.16 (1.06–1.27)

*ASD* Autism spectrum disorders, *GDD* Global developmental delay, *SPT* symbolic play test, *DLD* Developmental language disorder

### Exploration of the optimal cut-off value for SPT to identify two groups

**Fig. 1:**
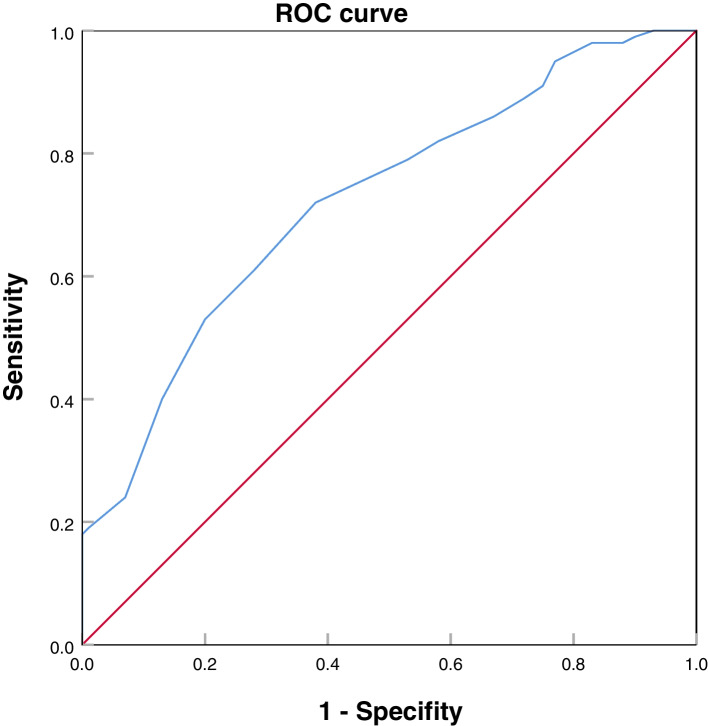
ROC curve of SPT screening for ASD without GDD

SPT scores were used as the test variable. Positive results of DSM-V and ADOS-2 were used as the state variable. Then ROC curve of the SPT score was plotted. The optimal cut-off value of SPT was determined by calculating the boundary point corresponding to the maximum of the Youden index. ROC curve showed that when the cut-off value of the SPT was 8.5, the largest area under the ROC curve was 0.723 (95% *CI*: 0.654 ~ 0.793, *P* < 0.001), and the sensitivity and specificity for the diagnosis of ASD without GDD were 0.720 and 0.620 respectively. Therefore, SPT had the best discriminatory effect on ASD without GDD and DLD when the SPT score is 8–9 (Fig. 1).

## Discussion

Early intervention can improve the core symptoms of children with ASD [20], and early detection is the key to achieving early intervention [33]. However, the age of diagnosis of ASD is still relatively late. A U.S. survey showed that the average age at diagnosis of ASD was 4.2 years [34]. The early identification and diagnosis of ASD lag behind in China [35], and the average age at diagnosis was 4.5 years [36].

SPT is a screening tool for young children in the early language stage, with a short test time and a simple method. It has been validated in English-speaking children. However, the assessment can be adapted to other cultures, such as Chinese and Japanese cultures, where chopsticks and bowls rather than spoons, forks, and knives are used as eating utensils [37].

This study indicates that SPT equivalent age was lower than the chronological age of the two groups. Therefore, SPT can assist in the screening of children with language developmental delay and ASD [6, 24]. The symbolic ability of the ASD without GDD group was significantly lower than that of the DLD group, a finding consistent with previous studies [38, 39]. There are statistically significant differences in the Language function, Personal-social function and Adaptive behavior function variables in the 18–24 months and 25–31 months groups. Statistically significant differences are also observed in the Fine motor function and Adaptive behavior function variables in the ≥32 months group. Logistic regression analysis showed that there was a difference in SPT equivalent age between DLD and ASD without GDD, independent of sex, age, and DQs of the five domains of CNBS-R2016. ROC curve showed that when the cut-off value of the SPT was 8–9, the largest area under the ROC curve was 0.723. It suggests that children with ASD still have difficulties in the symbolic game even without GDD. Symbolic play is an important behavior in early childhood development, which is closely related to social communication and cognitive development [40]. The earliest core symptoms of ASD were usually impairments in social interaction such as imaginary games and impairments in communication, rather than rigid repetitive movements or specific interests [41]. Other studies suggested that children with ASD have rigid and repetitive behaviors, lack complexity and diversity, and are difficult to understand the connection between objects. Moreover, they often show defects in pretend games and have less playfulness [42].

Prospective studies found that SPT is associated with developmental level or intelligence; ASD and developmental level both affect symbolic ability [5, 43]. However, few studies have explored the relationship between SPT and ASD independently of developmental level, and the optimal cut-off value for SPT to identify ASD.

### Limitations

The limitations of this study include the following aspects. First, the severity of ASD symptoms was not grouped. Therefore, it is difficult to further determine the application value of the SPT in different severities of ASD without GDD. Second, lack of neurotypical control samples. Setting a neurotypical control group may reduce the influence of confounding factors. Finally, we should also explore the discriminative role of SPT in children with ASD and GDD versus children with GDD alone, as the sample was comprised of children with ASD without GDD, the findings of this study may not be applicable to the entire ASD population.

## Conclusions

To sum up, symbolic play ability in ASD children is worse than that of DLD children at comparable development levels. In addition, SPT had the best discriminatory effect on ASD without GDD and DLD when the SPT score is 8–9. Our results, therefore, highlight the importance of assessing symbolic play. These findings may facilitate better identification of individuals at risk and the development of effective interventions to help children with ASD in China. Thus, SPT should be routinely used to screen for ASD in children who present with language delay without obvious developmental problems, and SPT should be used as an early warning signal of ASD to identify children suspected of ASD more sensitive and quickly so that they can be referred to hospitals at a higher level for comprehensive evaluation and diagnosis, and win the time of early intervention.

## Data Availability

The data sets generated and/or analyzed during the current study are not publicly available due to institutional policy but are available from the corresponding author on reasonable request.
